# Feasibility of Electronic Health Record Assessment of 6 Pediatric Type 1 Diabetes Self-management Habits and Their Association With Glycemic Outcomes

**DOI:** 10.1001/jamanetworkopen.2021.31278

**Published:** 2021-10-28

**Authors:** Joyce M. Lee, Andrea Rusnak, Ashley Garrity, Emily Hirschfeld, Inas H. Thomas, Michelle Wichorek, Jung Eun Lee, Nicole A. Rioles, Osagie Ebekozien, Sarah D. Corathers

**Affiliations:** 1 Susan B. Meister Child Health Evaluation and Research Center (CHEAR), University of Michigan, Ann Arbor; 2 Pediatric Endocrinology, University of Michigan, Ann Arbor; 3 Brehm Center for Diabetes Research, University of Michigan, Ann Arbor; 4 Caswell Diabetes Institute, Ann Arbor, Michigan; 5 T1D Exchange, Boston, Massachusetts; 6 Division of Endocrinology, Cincinnati Children’s Hospital Medical Center, University of Cincinnati College of Medicine, Cincinnati, Ohio

## Abstract

**Importance:**

A low-burden electronic health record (EHR) workflow has been devised to systematize the collection and validation of 6 key diabetes self-management habits: (1) checks glucose at least 4 times/day or uses continuous glucose monitor (CGM); (2) gives at least 3 rapid-acting insulin boluses per day; (3) uses insulin pump; (4) delivers boluses before meals; (5) reviewed glucose data since last clinic visit, and (6) has changed insulin doses since the last clinic visit.

**Objective:**

To describe the performance of these habits and examine their association with hemoglobin A_1c_ (HbA_1c_) levels and time in range (TIR).

**Design, Setting, and Participants:**

This cross-sectional study included individuals with known type 1 diabetes who were seen in a US pediatric diabetes clinic in 2019.

**Main Outcomes and Measures:**

Habit performance, total habit score (sum of 6 habits per person), HbA_1c_ levels, and TIR.

**Results:**

Of 1344 patients, 1212 (609 [50.2%] males; 66 [5.4%] non-Hispanic Black; 1030 [85.0%] non-Hispanic White; mean [SD] age, 15.5 [4.5] years) were included, of whom 654 (54.0%) were using CGM and had a TIR. Only 105 patients (8.7%) performed all 6 habits. Habit performance was lower among older vs younger patients (age ≥18 years vs ≤12 years: 17 of 411 [4.1%] vs 57 of 330 [17.3%]; *P* < .001), Black vs White patients (3 [4.5%] vs 95 [9.2%]; *P* < .001), those with public vs private insurance (14 of 271 [5.2%] vs 91 of 941 [9.7%]; *P* < .001), and those with lower vs higher parental education levels (<college degree vs ≥college degree: 35 of 443 [7.9%] vs 66 of 574 [11.5%]; *P* < .001). After adjustment for demographic characteristics and disease duration, for every 1-unit increase in total habit score, we found a mean (SE) 0.6% (0.05) decrease in HbA_1c_ among all participants and a mean (SE) 2.86% (0.71) increase in TIR among those who used CGMs. Multiple regression models revealed that performing each habit was associated with a significantly lower HbA_1c_ level (habit 1: –0.16% [95% CI, –1.91% to –1.37%]; habit 2: –1.01% [–1.34% to –0.69%]; habit 3: –0.71% [95% CI, –0.93% to –0.49%]; habit 4: –0.97% [95% CI, –1.21% to –0.73%]; habit 5: –0.44% [95% CI, –0.71% to –0.17%]; habit 6: –0.75% [95% CI, –0.96% to –0.53%]; all *P* < .001). There were differences in HbA_1c_ according to race, insurance, and parental education, but these associations were attenuated with the inclusion of the 6 habits, which had more robust associations with HbA_1c_ levels than the demographic characteristics.

**Conclusions and Relevance:**

These findings suggest that a focus on increasing adherence to the 6 habits could be critical for improving disparities in glycemic outcomes; these metrics have been adopted by the Type 1 Diabetes Exchange Quality Improvement Collaborative for continuous quality improvement.

## Key Points

**Question:**

What metrics of diabetes self-management behaviors collected as part of the clinical care workflow are associated with glycemic outcomes?

**Findings:**

In this cross-sectional study of 1212 patients with type 1 diabetes receiving care at a pediatric diabetes clinic, 6 patient-level habits associated with hemoglobin A_1c_ (HbA_1c_) levels were developed and validated. For every 1-unit increase in total habit score, a 0.6% decrease in HbA_1c_ was observed; when the 6 habits were incorporated in multiple regression models, associations of age, race, insurance, and parent education with HbA_1c_ levels were attenuated, and the habits had more robust associations with HbA_1c_ levels than demographic characteristics.

**Meaning:**

These findings suggest that adopting the 6 habits to support quality improvement interventions could produce more equitable outcomes.

## Teaser

This cross-sectional study describes the use of an electronic health record (EHR) workflow to assess 6 pediatric type 1 diabetes self-management habits and examines the association of these habits with hemoglobin A_1c_ (HbA_1c_) levels and time in range.

## Introduction

Despite the landmark findings of the 1993 Diabetes Control and Complications Trial, demonstrating that frequent blood glucose (BG) monitoring and intensive insulin therapy were effective for improving hemoglobin A_1c_ (HbA_1c_) levels and delaying the onset and progression of microvascular complications in type 1 diabetes,^1^ there has been a translational gap in the achievement of optimal glycemic outcomes more than 25 years later. From 2016 to 2018,^2^ only 17% of children (<18 years old) and 21% of adults achieved American Diabetes Association (ADA) glycemic goals of HbA_1c_ levels of less than 7.5% (to convert to proportion of total hemoglobin, multiply by 0.01) or 58 mmol/mol^3^ for children and less than 7.0% or 53 mmol/mol^4^ for adults. In 2020, ADA set an even tighter glycemic goal of HbA_1c_ levels of less than 7.0% or 53 mmol/mol^5^ for children, which only 10.2% of patients achieved in 2020.^6^ Furthermore, significant racial, ethnic, and socioeconomic disparities in glycemic outcomes for racial and ethnic minority populations in the US (vs White populations) across the Type 1 Diabetes Exchange (T1DX) Research Registry^7^ and SEARCH study cohorts have been reported.^8^

Quality improvement (QI) methodology,^9^ supported by advances in health information technology, offers an opportunity to improve type 1 diabetes care and health disparities in glycemic outcomes. Simple metrics incorporated into the electronic health record (EHR) allow care teams to track processes and outcomes and tailor interventions. Patient self-management is an essential part of effective diabetes care, but unfortunately, key self-management habits associated with improved glycemic outcomes have not been consistently measured in the clinical setting using a structured, reportable format, limiting opportunities for conducting continuous QI.

We devised a relatively low-burden EHR workflow to systematically collect a series of 6 evidence-based diabetes self-management measures during clinic visits.^10,11,12,13^ The 6 habits were guided by the QI work of the T1DX Quality Improvement Collaborative (T1DX-QI),^15^ a multicenter QI collaborative that has been focused on improving glycemic outcomes through the key drivers of effective glucose monitoring (checks BG ≥4 times/d if not on a continuous glucose monitor [CGM] or uses CGM); effective insulin delivery (gives ≥3 rapid-acting insulin boluses per day; uses insulin pump; and delivers boluses before meals); and effective use of diabetes data (has reviewed glucose data for patterns at least once since the last clinic visit and has changed insulin doses at least once since the last clinic visit). Our objective was to describe demographic differences in the prevalence of performing the 6 habits and to examine the associations of these habits with HbA_1c_ levels and time in range (TIR), defined as the percentage of BG readings between 70 to 180 mg/dL (to convert to millimoles per liter, multiply by 0.0555) among those who use CGMs.^14^ Identifying an association between recorded self-management habits and glycemic outcomes would provide justification for the use of these metrics in clinical care and support continuous QI efforts.

## Methods

The C.S. Mott Children’s Hospital Pediatric Diabetes Program devised and implemented 6 self-management behavior metrics. An Epic physician-builder (J.M.L.) created EHR flowsheet items and tools to facilitate documentation at each clinic visit by the diabetes team (diabetes educator/endocrinologist), which went live in the EHR in April 2018. This project was filed as self-determined activities not regulated as human participant research with the University of Michigan Medical School institutional review board. Therefore, informed consent was not required. This study followed Strengthening the Reporting of Observational Studies in Epidemiology (STROBE) reporting guideline.

### Study Definitions

Given multiple type 1 diabetes visits per year, we extracted measures from the EHR from the last visit in 2019 for each unique patient, excluding patients diagnosed within 6 months. Table 1 shows the EHR flowsheet items, response options, and definitions of performing the habit. Habit 1 is checks BG at least 4 times/d if not using a CGM or uses CGM; habit 2, gives at least 3 rapid-acting insulin boluses per day; habit 3, uses insulin pump; habit 4, delivers boluses before meals; habit 5, reviewed glucose data for patterns at least once since the last clinic visit; and habit 6, changed insulin doses at least once since the last clinic visit. For patients using a CGM, the TIR was manually extracted through medical record review of the CGM device’s data summary report, which is imaged into the EHR for the clinic visit. Race and ethnicity were defined from the EHR as self-reported non-Hispanic Black, non-Hispanic White, and other, which included American Indian, Alaskan Native, or Native Hawaiian; Asian; Hispanic; multiracial; or unknown/did not wish to report.

**Table 1.** Habits, With Item Questions, Definitions, and Data Sources

Goal	Habit	Item question	Item response options	Definition of performing habit	Data source
Effective glucose monitoring	1	Blood glucose testing frequency on download	0 to ≥10 times	Checks blood glucose ≥4 times/d if not on a CGM or uses CGM	Based on clinician assessment of the 14-d diabetes device download for patients using downloadable meter, CGM, or pump or paper logbook; if device data are not available, patient or parent self-report is used
		Uses CGM	Yes or no		
Effective insulin delivery	2	Average No. of bolus insulin doses per day on download (for pump) and patient report (for MDI)	0 to ≥10 times	Gives ≥3 rapid-acting insulin boluses/d	
Effective insulin delivery	3	Type of intensive therapy	MDI or insulin pump therapy	Uses insulin pump	
Effective insulin delivery	4	Timing of insulin with meals	“At least several minutes before the meal,” “Immediately before the meal,” or “During or after the meal”	Delivers boluses before meals; response of “At least several minutes before the meal” or “Immediately before the meal”	Based on patient/parent self-report for most bolus uses
Effective use of diabetes data	5	Times blood glucose or insulin data were downloaded and reviewed for blood glucose patterns since the last diabetes clinic visit	0 to ≥10 times	Reviewed glucose data for patterns at least once since the last clinic visit	Based on patient or parent self-report
Effective use of diabetes data	6	Times insulin was adjusted by family or by diabetes team since the last diabetes clinic visit	0 to ≥10 times	Changed insulin doses at least once since the last clinic visit (initiated by family or clinic)	

Abbreviations: CGM, continuous glucose monitor; MDI, multiple daily injections.

### Statistical Analysis

#### Main Analyses

We performed similar analyses with HbA_1c_ levels and TIR as continuous variables. We assessed frequency of habit performance and conducted χ^2^ tests to look for demographic differences and performed *t* tests and linear regression to compare differences in HbA_1c_ and TIR by demographic characteristics and habits. We summed each habit performed into a total habit score out of a possible maximum of 6. We evaluated the association of HbA_1c_ levels TIR with the number of habits through a multiple regression model adjusting for demographic characteristics and diabetes duration. Finally, we ran multiple regression models looking at the association of HbA_1c_ and TIR with combinations of demographic characteristics, diabetes duration, and each habit. However, for TIR we only looked at 5 habits, given that by definition these patients were already performing habit 1.

#### Sensitivity Analyses

Because a subset of patients had 1 or more missing fields for the habits, we assumed that a missing habit was equivalent to not performing the habit, but we also conducted a series of sensitivity analyses looking at subsets of patients: those with no missing habits; those who did not use CGMs; and excluding patients using the MiniMed 670G insulin pump (Medtronic) in Auto Mode, which performs automatic dosing of insulin for high and low blood glucoses and therefore could affect HbA_1c_ levels independently. Furthermore, we ran models that adjusted for CGM (flash vs real-time) and insulin delivery method (multiple daily injections vs pump). All analyses in this study were 2-sided hypothesis tests, and *P* <.05 was considered statistically significant. Data analyses were completed with R version 3.6.2 (R Core Team).

## Results

Of the 1344 patients with type 1 diabetes seen in 2019, we excluded 57 individuals less than 180 days from diagnosis; 2 not yet requiring insulin; 9 missing an HbA_1c_ level; and 64 missing documentation of all 6 habits; leaving 1212 individuals (90.2% of the clinic population; 609 [50.2%] males; 66 [5.4%] non-Hispanic Black; 1030 [85.0%] non-Hispanic White; mean [SD] age, 15.5 [4.5]). For the TIR analysis, of the 718 patients who were using a CGM, we excluded 64 patients with missing TIR, leaving 654 individuals in the analysis.

Table 2 shows demographic characteristics and HbA_1c_ levels overall and by CGM use. Overall, 278 patients (22.9%) had an HbA1c level of less than 7.5%, and 50 (4.1%) had an HbA_1c_ level of less than 7.0%. There were significant differences in HbA_1c_ levels for older vs younger patients, Black vs White children, children with public vs private insurance, and children with parents with less than a college degree vs college degree or greater. No significant differences were seen for an HbA_1c_ level of less than 7%. eTable 1 in the Supplement shows TIR statistics for those who used CGMs, with 206 (31.5%) with TIR of at least 50%. eTable 2 in the Supplement shows characteristics by insulin delivery method (multiple daily injections vs pump).

**Table 2.** Demographic Breakdown and Glycemic Outcomes for the Overall Population and by CGM Use

Characteristic	All patients					Patients not using CGM					Patients using CGM				
	Mean HbA_1c_ level			HbA_1c_ <7.5%		Mean HbA_1c_ level			HbA_1c_ <7.5%		Mean HbA_1c_ level			HbA_1c_ <7.5%	
	No. (%)	Mean (SD)	*P* value	No. (%)	*P* value	No. (%)	Mean (SD)	*P* value	No. (%)	*P* value	No. (%)	Mean (SD)	*P* value	No. (%)	*P* value
Overall	1212 (100)	8.9 (1.9)	NA	278 (22.9)	NA	494 (40.8)	9.8 (2.1)	NA	57 (4.7)	NA	718 (59.2)	8.3 (1.5)	NA	221 (18.2)	NA
Sex															
Male	609 (50.2)	8.9 (2.0)	.53	139 (22.8)	.89	251 (20.7)	10.0 (2.2)	.09	30 (2.5)	.06	358 (29.5)	8.2 (1.5)	.27	109 (9.0)	.33
Female	603 (49.8)	8.8 (1.9)		139 (23.1)		243 (20.0)	9.6 (2.1)		27 (2.2)		360 (29.7)	8.4 (1.6)		112 (9.2)	
Age, y															
0-12	330 (27.2)	8.4 (1.5)	<.001	81 (24.5)	.12	99 (8.2)	9.6 (1.7)	.52	6 (0.5)	.41	231 (19.1)	8.0 (1.1)	.002	75 (6.2)	.10
13-17	471 (38.9)	9.0 (2.0)		97 (20.6)		182 (15.0)	10.0 (2.2)		17 (1.4)		289 (23.8)	8.4 (1.5)		80 (6.6)	
≥18	411 (33.9)	9.1 (2.2)		100 (24.3)		213 (17.6)	9.8 (2.3)		34 (2.8)		198 (16.3)	8.4 (1.9)		66 (5.4)	
Race and ethnicity^a^															
White	1030 (85.0)	8.8 (1.9)	<.001	244 (23.7)	.03	391 (32.3)	9.6 (2.1)	.002	50 (4.1)	.58	639 (52.7)	8.3 (1.5)	.33	195 (16.1)	.34
Black	66 (5.4)	10.2 (2.2)		5 (7.6)		46 (3.8)	10.7 (2.2)		1 (0.1)		20 (1.7)	8.8 (1.3)		4 (0.3)	
Other	116 (9.6)	9.2 (2.3)		29 (25.0)		57 (4.7)	10.0 (2.3)		6 (0.5)		59 (4.9)	8.3 (2.0)		22 (1.8)	
Primary insurance															
Private	941 (77.6)	8.6 (1.8)	<.001	239 (25.4)	.67	312 (25.7)	9.5 (2.1)	<.001	42 (3.5)	.76	629 (51.9)	8.3 (1.5)	.26	197 (16.3)	.48
Public	271 (22.4)	9.6 (2.2)		39 (14.4)		182 (15.0)	10.2 (2.2)		15 (1.2)		89 (7.3)	8.5 (1.7)		24 (2.0)	
Parental education															
≥College degree	574 (47.4)	8.4 (1.7)	<.001	169 (29.4)	.30	155 (12.8)	9.3 (1.9)	.002	22 (1.8)	.48	419 (34.6)	8.1 (1.4)	<.001	147 (12.1)	.43
<College degree	443 (36.6)	9.4 (2.1)		63 (14.2)		246 (20.3)	10.1 (2.2)		22 (1.8)		197 (16.3)	8.6 (1.6)		41 (3.4)	
Unknown	195 (16.1)	9.1 (2.1)		46 (23.6)		93 (7.7)	9.8 (2.2)		13 (1.1)		102 (8.4)	8.4 (1.7)		33 (2.7)	

Abbreviations: CGM, continuous glucose monitor; HbA_1c_, hemoglobin A_1c_; NA, not applicable.

SI conversion: To convert HbA_1c_ to proportion of total hemoglobin, multiply by 0.01.

^a^ Participants who self-reported race and ethnicity as Black and White were classified as non-Hispanic. Other includes American Indian, Alaskan Native, or Native Hawaiian; Asian; Hispanic; multiracial; or unknown/did not wish to report.

Table 3 shows the prevalence of habits for the population and by subgroup. Overall, the most performed was habit 2 (≥3 insulin boluses/d; 1071 [88.4%]), followed by habit 1 (982 [81.0%]), habit 4 (delivering bolus before meals; 866 [71.5%]), and habit 3 (using an insulin pump; 731 [60.3%]). Habits 5 and 6 (reviewing data between visits [240 (19.8%)] and changing insulin doses between visits [452 (37.3%)], respectively) were the least commonly performed habits. We found significant differences in the percentage of patients who performed each habit by age, race, insurance, and parental education. There were lower rates of habit performance among older compared with younger patients (age ≥18 years vs ≤12 years: 17 of 411 [4.1%] vs 57 of 330 [17.3%]; *P* < .001), Black compared with White patients (3 [4.5%] vs 95 [9.2%]; *P* < .001), patients with public vs private insurance (14 of 271 [5.2%] vs 91 of 941 [9.7%]; *P* < .001), and patients with parents with less than a college degree vs parents with a college degree or greater (<college degree vs ≥college degree: 35 of 443 [7.9%] vs 66 of 574 [11.5%]; *P* < .001). Only 105 patients (8.7%) performed all 6 habits.

**Table 3.** Percentage of Patients Who Perform Each Habit by Age, Sex, Race, Insurance, and Parental Education

Characteristic	Habit 1a, No. (%)^a^	*P* value	Habit 1b, No. (%)^a^	*P* value	Habit 1, No. (%)	*P* value	Habit 2, No. (%)	*P* value	Habit 3, No. (%)	*P* value	Habit 4, No. (%)	*P* value	Habit 5, No. (%)	*P* value	Habit 6, No. (%)	*P* value	All habits No. (%)	*P* value
Overall	262 (53.0)	NA	720 (59.4)	NA	982 (81.0)	NA	1071 (88.4)	NA	731 (60.3)	NA	866 (71.5)	NA	240 (19.8)	NA	452 (37.3)	NA	105 (8.7)	NA
Age, y																		
0-12	78 (78.8)	.04	231 (70.0)	<.001	309 (93.6)	<.001	319 (96.7)	<.001	196 (59.4)	.48	267 (80.9)	.07	108 (32.7)	<.001	179 (54.2)	<.001	57 (17.3)	<.001
13-17	84 (46.2)		291 (61.8)		375 (79.6)		402 (85.4)		296 (62.8)		334 (70.9)		77 (16.3)		158 (33.5)		31 (6.6)	
≥18	100 (46.9)		198 (48.2)		298 (72.5)		350 (85.2)		239 (58.2)		265 (64.5)		55 (13.4)		115 (28.0)		17 (4.1)	
Sex																		
Male	136 (54.6)	.49	359 (58.9)	.79	496 (81.4)	.85	539 (88.5)	.48	358 (58.8)	.44	434 (71.3)	.51	118 (19.4)	.81	220 (36.1)	.38	55 (9.0)	.72
Female	125 (51.4)		361 (59.9)		486 (80.6)		532 (88.2)		373 (61.9)		432 (71.6)		122 (20.2)		232 (38.5)		50 (8.3)	
Race and ethnicity^b^																		
Black	23 (50.0)	.03	20 (30.3)	<.001	43 (65.2)	<.001	54 (81.8)	.09	26 (39.4)	<.001	53 (80.3)	.03	9 (13.6)	.15	13 (19.7)	.003	3 (4.5)	.24
White	215 (55.0)		641 (62.2)		856 (83.1)		908 (88.2)		645 (62.6)		737 (71.6)		214 (20.8)		399 (38.7)		95 (9.2)	
Other	24 (42.1)		59 (50.9)		83 (71.6)		109 (94.0)		60 (51.7)		76 (65.5)		17 (14.7)		40 (34.5)		7 (6.0)	
Primary insurance																		
Private	174 (55.8)	<.001	631 (67.1)	<.001	805 (85.5)	<.001	838 (89.1)	.03	607 (64.5)	<.001	677 (71.9)	.03	200 (21.3)	.01	373 (39.6)	.001	91 (9.7)	.03
Medicaid	88 (48.4)		89 (32.8)		177 (65.3)		233 (86.0)		124 (45.8)		189 (69.7)		40 (14.8)		79 (29.2)		14 (5.2)	
Parental education																		
≥College degree	77 (49.7)	<.001	419 (73.0)	<.001	496 (86.4)	<.001	508 (88.5)	<0.001	387 (67.4)	<.001	461 (80.3)	<.001	144 (25.1)	<.001	250 (43.6)	<.001	66 (11.5)	<.001
<College degree	110 (44.7)		199 (44.9)		309 (69.8)		380 (85.8)		213 (48.1)		335 (75.6)		84 (19.0)		145 (32.7)		35 (7.9)	
Unknown	75 (80.6		102 (52.3)		177 (90.8)		183 (93.8)		131 (67.2)		70 (35.9)		12 (6.2)		57 (29.2)		4 (2.1)	

Abbreviations: CGM, continuous glucose monitor; NA, not applicable.

^a^ Habit 1a defined as checks blood glucose at least 4 times/day if not using a CGM; habit 1b, uses CGM.

^b^ Participants who self-reported race and ethnicity as Black and White were classified as non-Hispanic. Other includes American Indian, Alaskan Native, or Native Hawaiian; Asian; Hispanic; multiracial; or unknown/did not wish to report.

The Figure, A, shows that mean HbA_1c_ levels for individuals who performed each habit were significantly lower compared with those who did not perform the habit (eg, habit 1: 8.5% [1.7] vs 10.4% [2.3]; *P* < .001; habit 2: 8.8% [1.8] vs 10.9% [2.4]; *P* < .001). The Figure, B, shows the mean HbA_1c_ by total habit score. There was a significant negative trend between the number of habits and HbA1c (*P* < .001); for each 1-unit increase in total habit score, there was a 0.7% (8 mmol/mol) decrease in HbA_1c_ (mean [SE] of 0.6% [0.05] after adjustment for demographic characteristics and diabetes duration).

**Figure.**
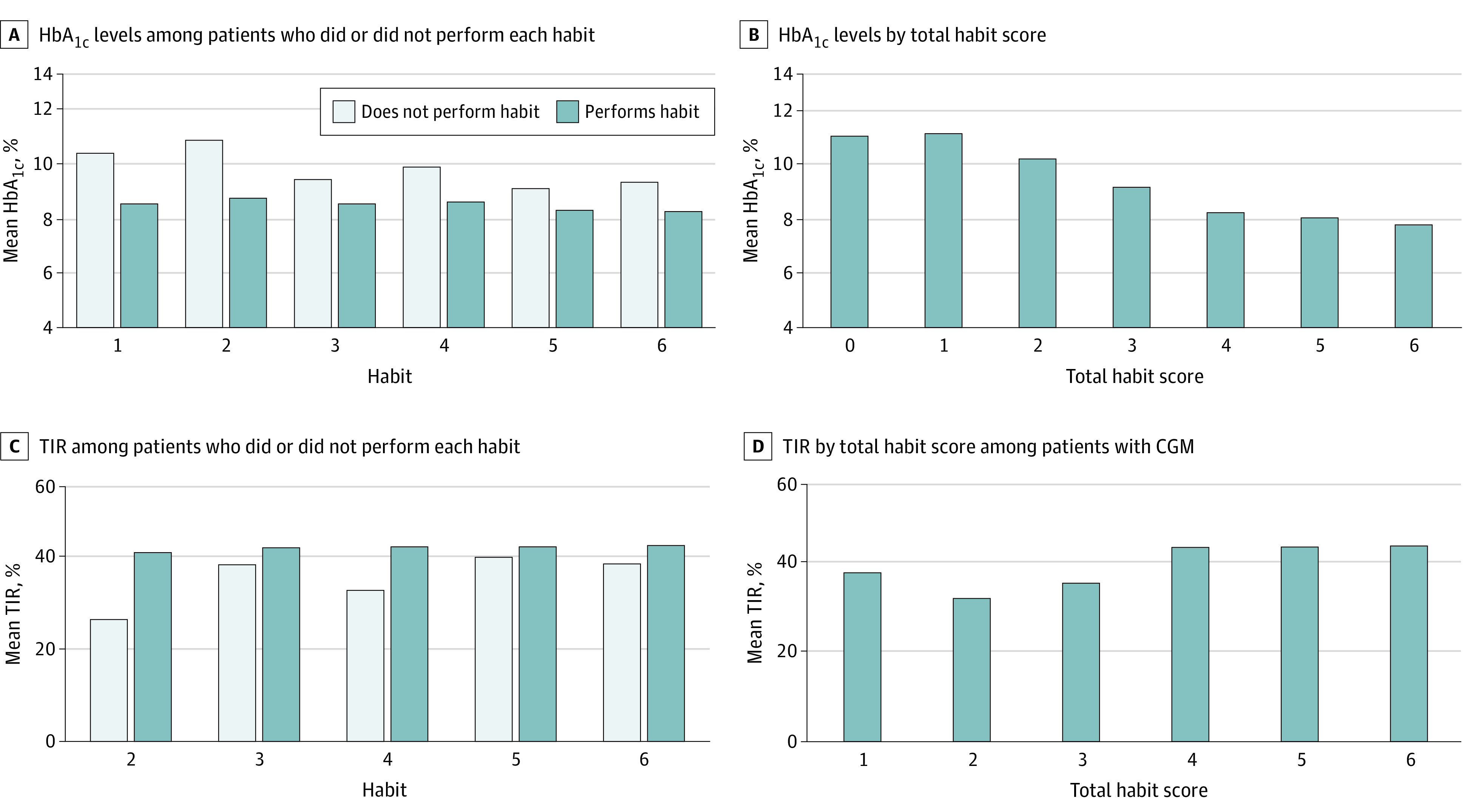
Hemoglobin A_1c _(HbA_1c_) Levels and Time in Range (TIR) by Each Habit and Total Habit Score A, Comparisons between those who performed and did not perform each habit were statistically significant (*P* < .001) for all habits. C, Comparisons between those who performed and did not perform habits 2, 3, 4, and 6 were statistically significant (habit 2, *P* = .006; habit 3, *P* = .03; habit 4, *P* < .001; and habit 6, *P* = .01). CGM indicates continuous glucose monitor. To convert HbA_1c_ levels to proportion of total hemoglobin, multiply by 0.01.

Figure, C, shows mean (SD) TIR for individuals who performed each habit, which was significantly higher compared with those who did not perform the habit, except for habit 5: habit 2 (40.9% [20.0] vs 26.2% [18.2]; *P* = .004), habit 3 (41.7% [20.1] vs 37.9% [19.8]; *P* = .03), habit 4 (42.0% [20.2] vs 32.5% [16.4]; *P* < .001), and habit 6 (42.5% [19.3] vs 38.3% [20.5]; *P* = .01). There was a significant positive trend between total habits and TIR; for each 1-unit increase in the total habit score, there was a 2.80% increase in TIR (mean [SE] of 2.86% [0.71] after adjustment for demographic characteristics and diabetes duration). The Figure, D shows average TIR by total habit score. eFigure 1 and eFigure 2 in the Supplement show similar figures across demographic subgroups.

Table 4 shows the multiple regression analyses with HbA_1c_ level as an outcome. With the demographic characteristics–only model, there were statistically significant differences in HbA_1c_ level by age, race, insurance, and parental education, with higher HbA_1c_ levels for Black vs White patients, older vs younger patients, patients with public vs private insurance, and patients with parents with less than a college degree vs parents with a college degree or more. With the 6 habits–only model (adjusted for duration of diabetes), performing each habit was associated with a lower HbA_1c_ level compared with those who did not perform the habit. In the combined model, including demographic characteristics and the habits (adjusted for duration of diabetes), associations with race, insurance, and parental education were attenuated compared with the demographic characteristics–only model, and the individual habits retained robust statistical significance.

**Table 4.** Association of Demographic Characteristics Only, Habits Only, and Habits Plus Demographic Characteristics with HbA_1c_ Levels for the Total Population and TIR for the Population Using CGMs

Characteristic	Demographic characteristics–only model^a^		Habits-only model^a^		Habits and demographic model^a^	
	Estimated change (95% CI)	*P* value	Estimated change (95% CI)	*P* value	Estimated change (95% CI)	*P* value
**HbA_1c_ level, %**						
Age, y						
0-12	0 [Reference]	NA	NA	NA	0 [Reference]	NA
13-17	0.53 (0.27 to 0.79)	<.001	NA	NA	–0.08 (–0.03 to 0.17)	.52
≥18	0.65 (0.37 to 0.92)	<.001	NA	NA	–0.37 (–0.67 to –0.08)	.01
Sex						
Male	0 [Reference]	NA	NA	NA	0 [Reference]	NA
Female	–0.07 (–0.27 to 0.14)	.54	NA	NA	–0.07 (–0.26 to 0.12)	.50
Race and ethnicity^b^						
White	0 [Reference]	NA	NA	NA	0 [Reference]	NA
Black	1.07 (0.60 to 1.54)	<.001	NA	NA	0.9 (0.48 to 1.32)	<.001
Other	0.26 (–0.10 to 0.62)	.15	NA	NA	0.11 (–0.21 to 0.43)	.52
Primary insurance						
Private	0 [Reference]	NA	NA	NA	0 [Reference]	NA
Public	0.67 (0.40 to 0.93)	<.001	NA	NA	0.48 (0.25 to 0.71)	<.001
Parental education						
≥College degree	0 [Reference]	NA	NA	NA	0 [Reference]	NA
<College degree	0.71 (0.47 to 0.95)	<.001	NA	NA	0.54 (0.32 to 0.75)	<.001
Unknown	0.45 (0.15 to 0.76)	.004	NA	NA	0.18 (–0.12 to 0.47)	.25
Habit 1						
Does not perform	NA	NA	0 [Reference]	NA	0 [Reference]	NA
Performs	NA	NA	–1.88 (–2.14 to –1.32)	<.001	–0.16 (–1.91 to –1.37)	<.001
Habit 2						
Does not perform	NA	NA	0 [Reference]	NA	0 [Reference]	NA
Performs	NA	NA	–1.13 (–1.46 to –0.79)	<.001	–1.01 (–1.34 to –0.69)	<.001
Habit 3						
Does not perform	NA	NA	0 [Reference]	NA	0 [Reference]	NA
Performs	NA	NA	–0.96 (–1.18 to –0.74)	<.001	–0.71 (–0.93 to –0.49)	<.001
Habit 4						
Does not perform	NA	NA	0 [Reference]	NA	0 [Reference]	NA
Performs	NA	NA	–0.93 (–1.16 to –0.69)	<.001	–0.97 (–1.21 to –0.73)	<.001
Habit 5						
Does not perform	NA	NA	0 [Reference]	NA	0 [Reference]	NA
Performs	NA	NA	–0.62 (–0.89 to –0.34)	<.001	–0.44 (–0.71 to –0.17)	<.001
Habit 6						
Does not perform	NA	NA	0 [Reference]	NA	0 [Reference]	NA
Performs	NA	NA	–0.93 (–1.15 to –0.71)	<.001	–0.75 (–0.96 to –0.53)	<.001
**TIR for 654 patients using CGM, %**						
Age, y						
0-12	0 [Reference]	NA	NA	NA	0 [Reference]	NA
13-17	–2.58 (–6.18 to 1.03)	.16	NA	NA	–1.41 (–5.17 to 8.78)	.46
≥18	1.48 (–2.60 to 5.56)	.48	NA	NA	3.75 (–0.92 to 8.42)	.12
Sex						
Male	0 [Reference]	NA	NA	NA	0 [Reference]	NA
Female	1.32 (–1.76 to 4.39)	.40	NA	NA	1.68 (–1.38 to 4.74)	.28
Race and ethnicity^b^						
White	0 [Reference]	NA	NA	NA	0 [Reference]	NA
Black	3.25 (–5.70 to 12.20)	.48	NA	NA	2.62 (–6.27 to 11.50)	.56
Other	–0.75 (–6.40 to 4.91)	.80	NA	NA	–0.24 (–5.72 to 5.24)	.93
Primary insurance						
Private	0 [Reference]	NA	NA	NA	0 [Reference]	NA
Public	0.8 (–4.24 to 5.84)	.76	NA	NA	–1.49 (–6.22 to 3.25)	.54
Parental education						
≥College degree	0 [Reference]	NA	NA	NA	0 [Reference]	NA
<College degree	–5.60 (–9.32 to –1.89)	.003	NA	NA	–4.95 (–8.44 to –1.46)	.01
Unknown	–2.10 (–6.90 to 2.70)	.39	NA	NA	0.66 (–4.40 to 5.72)	.80
Habit 2						
Does not perform	NA	NA	0 [Reference]	NA	0 [Reference]	NA
Performs	NA	NA	14.61 (4.64 to 24.57)	.004	13.88 (3.91 to 23.85)	.006
Habit 3						
Does not perform	NA	NA	0 [Reference]	NA	0 [Reference]	NA
Performs	NA	NA	4.29 (0.81 to 7.80)	.02	4.28 (0.77 to 7.80)	.02
Habit 4						
Does not perform	NA	NA	0 [Reference]	NA	0 [Reference]	NA
Performs	NA	NA	6.78 (3.08 to 10.00)	<.001	7.41 (3.53 to 11.29)	<.001
Habit 5						
Does not perform	NA	NA	0 [Reference]	NA	0 [Reference]	NA
Performs	NA	NA	1.88 (–1.68 to 5.44)	.30	1.72 (–1.89 to 5.34)	.35
Habit 6						
Does not perform	NA	NA	0 [Reference]	NA	0 [Reference]	NA
Performs	NA	NA	3.67 (0.55 to 6.80)	.02	3.70 (0.55 to 6.84)	.02

Abbreviations: CGM, continuous glucose monitor; HbA_1c_, hemoglobin A_1c_; NA, not applicable.

^a^ All models are adjusted for duration of diabetes.

^b^ Participants who self-reported race and ethnicity as Black and White were classified as non-Hispanic. Other includes American Indian, Alaskan Native, or Native Hawaiian; Asian; Hispanic; multiracial; or unknown/did not wish to report.

The results of the multiple regression analyses with TIR as an outcome variable among the subpopulation using a CGM appear in Table 4 as well. With the demographic characteristics–only model, the only significant association with TIR was lower TIR for children with parents with less than a college degree vs greater. With the habits-only model (which included only habits 2 through 6 because, by definition, patients were already performing habit 1) performing habits 2, 3, 4, and 6 was significantly associated with higher TIR. In the combined model including demographic characteristics and all 5 habits, habits 2, 3, 4, and 6 remained statistically associated with higher TIR, and statistical associations remained but were attenuated for parental education. Our findings were consistent in our sensitivity analyses for subsets of individuals, including those who had all 6 habits filled out (eTable 3 in the Supplement), those who did not use CGMs (eTable 4 in the Supplement), those not using the MiniMed 670G system in Auto Mode (eTable 5 in the Supplement), and when we adjusted for flash vs real-time CGM (eTable 6 in the Supplement) among those who used CGMs.

## Discussion

For the population of patients seen at our diabetes center, we found that the 6 habits can be efficiently and reliably collected as discrete data elements in routine clinical care and that performance of each of the habits and the total habit score are significantly associated with improved glycemic outcomes, regardless of age, race, sex, insurance status, and parental education. Key innovations of this work are the simplicity of the metrics with operational definitions, the use of EHR tools for tracking and measurement, and the incorporation of metrics into the clinical workflow, which will permit their expansion across a more geographically and demographically diverse group of T1DX-QI diabetes centers. Type 1 diabetes requires a bewildering number of tasks, but the 6 habits give clinicians and patients a set of simple heuristics on which to focus and act in real-time through shared decision-making and personalized interventions. Clear and consistent goals can be set around the habits while working toward the goal of improving glycemic management.

Our finding that associations with race, insurance, and parental education were attenuated after inclusion of the habits to the model and the fact that individual habits (ie, 1-4 and 6) had more robust associations with HbA_1c_ than demographic characteristics would suggest that a focus on the 6 habits is critical for reducing disparities in health outcomes. We hypothesize that clinicians and clinics that focus on improving adoption of the habits could reduce differences in glycemic outcomes for populations adversely affected by social determinants of health, defined as the “conditions in which people are born, grow, live, work, and age”^16^ that impact health outcomes.^17^ As a result, the T1DX-QI (34 US pediatric and adult centers) has a data roadmap that will require participating centers to measure the adoption of the 6 habits, and the network has established a 10-step framework for reducing health disparities in glycemic outcomes.^18^

We note that at least 3 of the habits directly relate to having access to technology (diabetes devices, computer/mobile devices, and internet access). Wearing a CGM facilitates greater awareness of BG patterns, wearing a pump facilitates more frequent and timely administration of insulin, and having a computer or mobile phone with internet access facilitates seamless data downloads from diabetes devices to review and adjust insulin. Unfortunately, there are stark racial disparities in device use in the United States, with the T1DX-QI recently reporting dramatically lower rates of CGM use for non-Hispanic Black patients (17%) compared with White (40%) and Hispanic (37%) patients, and dramatically lower rates of insulin pump use for non-Hispanic Black patients (41%) compared with White (60%) and Hispanic (56%) patients.^19^ Possible racial discrimination or bias, suboptimal insurance coverage, and high out-of-pocket costs are key structural barriers that must be addressed; the T1DX-QI has already begun some of this work in the areas of CGM and pump adoption.^20,21^

Surprisingly, less than 9% of the total clinic population performed all habits, illuminating the fundamental challenge of effective self-management. The biggest opportunities for improvement were the habits related to data review and insulin dose changes. Although habit 5 (data review) was individually associated with lower HbA_1c_ levels, its association with HbA_1c_ levels was not significant after inclusion of the other habits in the regression model. These findings differ from a previous study by Wong et al,^12^ which reported that patients who downloaded and reviewed their data at least 4 times a year had lower mean(SD) HbA_1c_ levels than those who did not routinely review their data (7.8% [1.4] vs 8.6% [1.7]). However, their study asked about data review only and did not measure insulin dose changes, which may account for these findings.

Habits 5 and 6 are linked, as the purpose of data review is to identify patterns of hypoglycemia or hyperglycemia that would signal the need for changes in behavior and/or insulin management. Data review alone without follow-up action would not be expected to affect glucose levels, but we elected to keep the data review measure as a component of the habits because we believe it is a critical self-management skill that should be regularly performed, particularly for children who need frequent adjustments due to growth and puberty. Although the diabetes team often recommends changes at visits or when families call for assistance, families are ultimately best served if they are empowered to independently review data and make adjustments.

Our finding that older age was associated with lower uptake of the 6 habits and a higher HbA_1c_ levels corroborates the need to focus on habits for the adolescent and young adult population as well, given the dramatic increase in HbA_1c_ levels that has been described in both North American and European cohorts for this age group.^2^ Given the potential glycemic benefit of adding even just 1 additional habit, clinicians could partner with patients and their adult caregivers and social support networks to share the burdens of complex self-management tasks.

With increasing use of CGM, TIR is the desired standard for assessing glycemic management given that it is a direct measure of glucose that can more accurately capture periods of hyperglycemia and hypoglycemia. We are unaware of studies that have looked at the association of the 6 habits and the total habit score with TIR. Our analysis of habits 2 through 6 corroborates the finding that the higher the number of habits performed, the greater the TIR. In the demographic characteristics–only model, we were surprised to find no associations with TIR by race or insurance, although having a parent with less than a college degree compared with a college degree or greater was associated with a less favorable TIR. This may be due to the small numbers of Black patients and patients with public insurance who had a CGM.

We acknowledge that numerous studies have described racial disparities in glycemic outcomes and associations of certain self-management behaviors,^2,7^ but these were collected for a subset of research participants who may not be representative. The innovation for this analysis was the development and validation of evidence-based self-management metrics against glycemic outcomes, and the formulation of a package of 6 habits that can be measured using sustainable and real-time clinical workflows in the EHR across the population, helping to realize the broad vision of a learning health system for type 1 diabetes.^22^

### Limitations

We acknowledge limitations of this study. First, there were missing data on habit performance for a subset of the population, which can happen with any real-world clinical workflow, but we successfully captured metrics for more than 90% of the population. Second, there were patients using automated systems, but our sensitivity analyses without those users demonstrated similar findings. As the use of automated systems increases, we believe that the 6 habits will remain relevant considering that individuals still must enter carbohydrate intake, deliver boluses before meals, and periodically adjust insulin. Third, the association of habits with TIR was not as robust as that with HbA_1c_ levels, possibly because patients who used CGMs had a lower mean HbA_1c_ level and because of the greater variability of glucose levels compared with HbA_1c_ levels.^23^ Fourth, we acknowledge that there are additional unmeasured variables that we have not accounted for and that may substantially affect the adoption of the 6 habits, such as social determinants of health (food insecurity, disconnected utilities, housing stability, childcare, health care affordability, transportation, literacy, and safety)^24,25^ as well as household/family structure (parental employment, income, education, and marital status)^26,27,28^ and other social supports.

## Conclusions

In this study, patients who performed even 1 of the 6 habits of type 1 diabetes self-management had lower HbA_1c_ levels and a greater percentage of TIR. The associations between these habits were more robust than those between demographic characteristics and glycemic control, suggesting that adoption of the 6 habits could be a critical tool for improving disparities in glycemic outcomes. As these simple and sustainable metrics are adopted across the T1DX-QI collaborative,^15^ we will have the capacity to assess greater generalizability of the metrics and develop collaborative multicenter interventions. We anticipate that these 6 habits will become universal across diabetes centers with the goal of increasing the overall proportion of individuals who perform them and ultimately closing the racial, socioeconomic, and educational gaps in habit performance and HbA_1c_ level.

## Notes

Supplement. **eTable 1.** Demographic Breakdown, Glycemic Outcomes, and Time in Range for 654 Patients Using CGM **eTable 2.** Demographic Breakdown and Glycemic Outcomes for Individuals on Multiple Daily Injections (MDI) vs Pump Therapy **eFigure 1. **Average HbA_1c_ Level by Habit Performance for Demographic Subgroups **eFigure 2. **Average HbA_1c_ Level and Time In Range (TIR) by Total Habit Score by Demographic Subgroups **eTable 3. **Association of Demographic Variables Only, Habits Only, and Habits Plus Demographics with HbA_1c_ for 852 Patients with No Missing Habits and TIR for 506 Patients Using CGM with No Missing Habits **eTable 4. **Association of Demographic Variables Only, Habits Only, and Habits Plus Demographics with HbA_1c_ for 494 Patients Not Using CGM **eTable 5. **Association of Demographic Variables Only, Habits Only, and Habits Plus Demographics with HbA_1c_ for 749 Patients Using Pumps, Excluding Auto Mode Pumps **eTable 6. **Association of Demographic Variables Only, Habits Only, and Habits Plus Demographics with HbA_1c_ for 692 Patients Using CGMs and Time in Range for 634 Patients Using CGMs, Adjusting for Flash vs Real-Time CGM
