# Errors in Abstract, Table 3, and Table 4

**DOI:** 10.1001/jamanetworkopen.2022.0001

**Published:** 2022-01-26

In the Original Investigation titled “Feasibility of Electronic Health Record Assessment of 6 Pediatric Type 1 Diabetes Self-management Habits and Their Association With Glycemic Outcomes,”^1^ published October 28, 2021, there were errors in the Abstract, Table 3, and Table 4. In the Abstract and Table 4, the coefficient for the association of habit 1 with hemoglobin A_1c_ levels should have read −1.65%. In Table 3, the number of male patients who performed habit 1a should have read 137. This article has been corrected.^1^
