# Supplementary material for: Feasibility of Electronic Health Record Assessment of 6 Pediatric Type 1 Diabetes Self-management Habits and Their Association With Glycemic Outcomes
Source: JAMA Netw Open. 2021 Oct 28;4(10):e2131278. doi: 10.1001/jamanetworkopen.2021.31278 (PMC8554640; doi:10.1001/jamanetworkopen.2021.31278)
Supplement: Supplement. — eTable 1. Demographic Breakdown, Glycemic Outcomes, and Time in Range for 654 Patients Using CGM eTable 2. Demographic Breakdown and Glycemic Outcomes for Individuals on Multiple Daily Injections (MDI) vs Pump Therapy eFigure 1. Average HbA1c Level by Habit Performance for Demographic Subgroups eFigure 2. Average HbA1c Level and Time In Range (TIR) by Total Habit Score by Demographic Subgroups eTable 3. Association of Demographic Variables Only, Habits Only, and Habits Plus Demographics with HbA1c for 852 Patients with No Missing Habits and TIR for 506 Patients Using CGM with No Missing Habits eTable 4. Association of Demographic Variables Only, Habits Only, and Habits Plus Demographics with HbA1c for 494 Patients Not Using CGM eTable 5. Association of Demographic Variables Only, Habits Only, and Habits Plus Demographics with HbA1c for 749 Patients Using Pumps, Excluding Auto Mode Pumps eTable 6. Association of Demographic Variables Only, Habits Only, and Habits Plus Demographics with HbA1c for 692 Patients Using CGMs and Time in Range for 634 Patients Using CGMs, Adjusting for Flash vs Real-Time CGM [file jamanetwopen-e2131278-s001.pdf]

Supplemental Online Content

Lee JM, Rusnak A, Garrity A, et al. Feasibility of electronic health record assessment of 6 pediatric type 1 diabetes self-management habits and their association with glycemic outcomes. *JAMA Netw Open.* 2021;4(10):e2131278. doi:10.1001/jamanetworkopen.2021.31278

eTable 1. Demographic Breakdown, Glycemic Outcomes, and Time in Range for 654 Patients Using CGM

eTable 2. Demographic Breakdown and Glycemic Outcomes for Individuals on Multiple Daily Injections (MDI) vs Pump Therapy

eFigure 1. Average HbA_{1c} Level by Habit Performance for Demographic Subgroups

eFigure 2. Average HbA_{1c} Level and Time In Range (TIR) by Total Habit Score by Demographic Subgroups

eTable 3. Association of Demographic Variables Only, Habits Only, and Habits Plus Demographics with HbA_{1c} for 852 Patients with No Missing Habits and TIR for 506 Patients Using CGM with No Missing Habits

eTable 4. Association of Demographic Variables Only, Habits Only, and Habits Plus Demographics with HbA_{1c} for 494 Patients Not Using CGM

eTable 5. Association of Demographic Variables Only, Habits Only, and Habits Plus Demographics with HbA_{1c} for 749 Patients Using Pumps, Excluding Auto Mode Pumps

eTable 6. Association of Demographic Variables Only, Habits Only, and Habits Plus Demographics with HbA_{1c} for 692 Patients Using CGMs and Time in Range for 634 Patients Using CGMs, Adjusting for Flash vs Real-Time CGM

This supplemental material has been provided by the authors to give readers additional information about their work.

© 2021 Lee JM et al. *JAMA Network Open.*

eTable 1. Demographic Breakdown, Glycemic Outcomes, and Time in Range for 654 Patients Using CGM

	**Mean HbA1c**			**HbA1c <7.5**		**Mean TIR (%)**		**TIR ≥50%**	
**CGM users with TIR data**	**n (%)**	**Mean (SD)**	***P* value**	**n (%)**	***P* value**	**Mean (SD)**	***P* value**	**n (%)**	***P* value**
**Overall**	654 (100)	8.2 (1.5)		215 (32.9)		40.5 (20.1)		206 (31.5)	
**Sex**									
Male	319 (48.8)	8.2 (1.4)	0.35	109 (16.7)	0.41	39.7 (19.7)	0.32	91 (28.5)	0.92
Female	335 (51.2)	8.3 (1.5)		106 (16.2)		41.3 (20.4)		115 (34.3)	
**Age Group, years**									
0-12	222 (33.9)	8.0 (1.2)	1.08	73 (11.2)	0.07	41.5 (18.4)	0.83	71 (32.0)	0.03
13-17	265 (40.2)	8.4 (1.5)		78 (11.9)		38.4 (20.0)		76 (28.7)	
18+	167 (25.5)	8.2 (1.8)		64 (9.8)		42.4 (22.1)		59 (35.3)	
**Race/Ethnicity**									
White Non-Hispanic	580 (88.7)	8.2 (1.4)	0.18	190 (29.1)	0.3	40.4 (19.9)	0.86	179 (30.9)	0.59
Black Non-Hispanic	20 (3.1)	8.8 (1.3)		4 (0.6)		43.0 (17.0)		7 (35.0)	
Other	54 (8.3)	8.2 (1.9)		21 (3.2)		40.4 (22.7)		20 (37.0)	
**Primary Insurance**									
Private	577 (88.2)	8.2 (1.4)	0.19	192 (29.4)	0.44	40.6 (19.9)	0.62	179 (31.0)	0.48
Public	77 (11.8)	8.5 (1.8)		23 (3.5)		39.4 (21.1)		27 (35.1)	
**Parental Education**									
College Degree or More	389 (59.5)	8.0 (1.4)	< 0.001	142 (21.7)	0.39	42.3 (20.0)	0.009	130 (33.4)	0.15
Less than College Degree	182 (27.8)	8.6 (1.6)		40 (6.1)		36.8 (20.0)		44 (24.2)	
Unknown	83 (12.7)	8.2 (1.6)		33 (5.0)		39.3 (19.8)		32 (38.6)	

© 2021 Lee JM et al. *JAMA Network Open.*

eTable 2. Demographic Breakdown and Glycemic Outcomes for Individuals on Multiple Daily Injections (MDI) vs Pump Therapy

	**Multiple Daily Injections**					**Pump (Auto Mode Pump excluded)**				
	**Mean HbA1c**			**HbA1c <7.5**		**Mean HbA1c**			**HbA1c <7.5**	
	**n (%)**	**Mean**	***P* value**	**n (%)**	***P* value**	**n (%)**	**Mean**	***P* value**	**n (%)**	***P* value**
**Overall**	419 (34.6)	9.5		67 (5.5)		749 (61.8)	8.6		199 (16.4)	
**Sex**										
Male	213 (17.6)	9.5	0.99	33 (2.7)	0.05	375 (30.9)	8.7	0.27	97 (6.8)	0.14
Female	206 (17.0)	9.5		34 (2.8)		374 (30.9)	8.5		102 (6.9)	
**Age Group, years**										
0-12	116 (9.6)	9.0	0.002	20 (1.7)	0.10	202 (16.7)	8.2	0.002	57 (4.7)	0.67
13-17	153 (12.6)	9.5		24 (2.0)		299 (24.7)	8.8		68 (5.6)	
18+	150 (12.4)	9.9		23 (1.9)		248 (20.5)	8.7		74 (6.1)	
**Race/Ethnicity**										
White Non-Hispanic	329 (27.1)	9.3	0.003	55 (4.5)	0.47	659 (54.4)	8.6	< 0.001	177 (14.6)	0.04
Black Non-Hispanic	37 (3.1)	10.4		3 (0.2)		29 (2.4)	9.9		2 (0.2)	
Other	53 (4.4)	10.0		9 (0.7)		61 (5.0)	8.6		20 (1.7)	
**Primary Insurance**										
Private	278 (22.9)	9.2	< 0.001	55 (4.5)	0.51	624 (51.5)	8.5	< 0.001	176 (6.8)	0.75
Public	141 (11.6)	10.1		12 (1.0)		125 (10.3)	9.3		23 (6.8)	
**Parental Education**										
College Degree or More	152 (12.5)	8.8	< 0.001	36 (3.0)	0.62	398 (32.8)	8.3	< 0.001	126 (10.4)	0.25
Less than College Degree	210 (17.3)	9.9		21 (1.7)		226 (18.6)	9.1		39 (3.2)	
Unknown	57 (4.7)	9.8		10 (0.8)		125 (10.3)	8.7		34 (2.8)	

© 2021 Lee JM et al. *JAMA Network Open.*

eFigure 1. Average HbA_{1c} Level by Habit Performance for Demographic Subgroups[a]

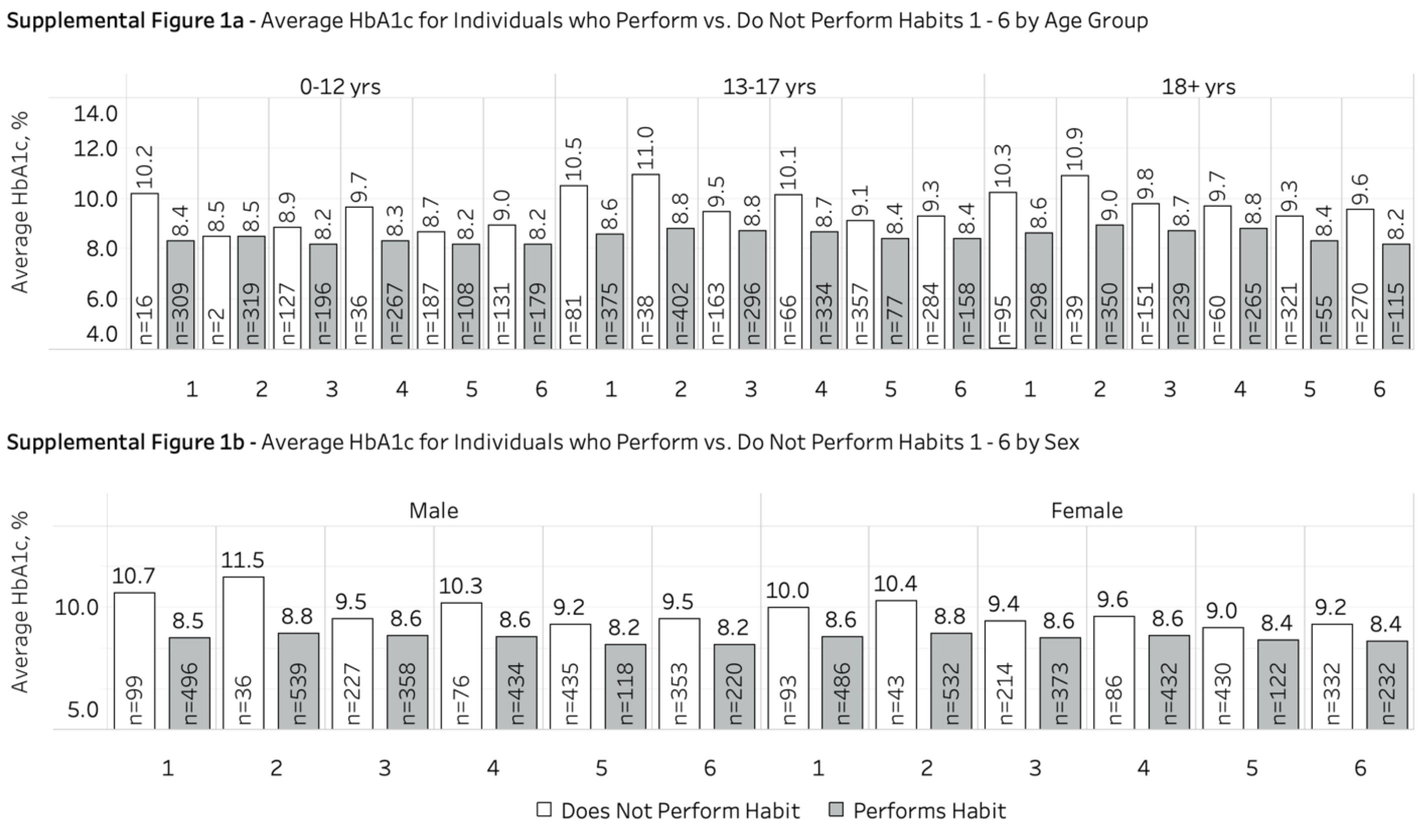

© 2021 Lee JM et al. *JAMA Network Open.*

Supplemental Figure 1c - Average HbA1c for Individuals who Perform vs. Do Not Perform Habits 1 - 6 by Race

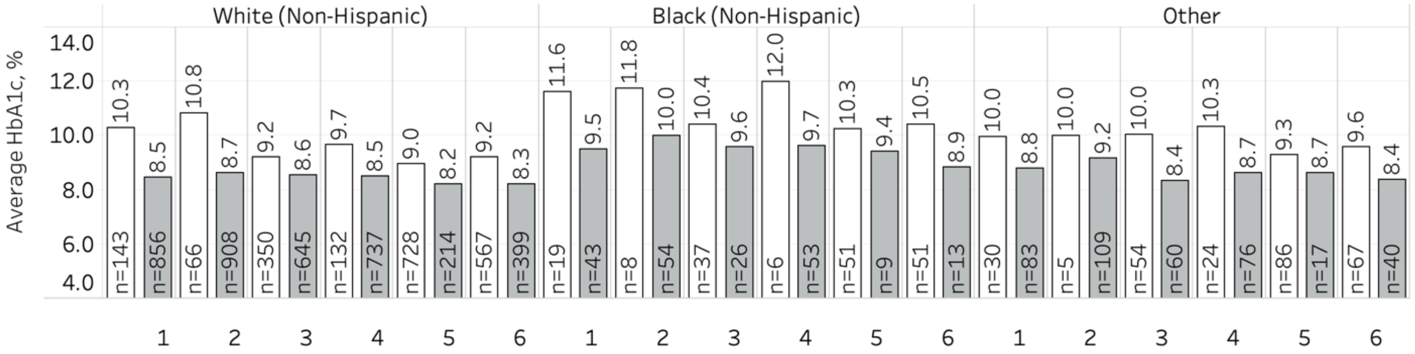

Supplemental Figure 1d - Average HbA1c for Individuals who Perform vs. Do Not Perform Habits 1 - 6 by Insurance

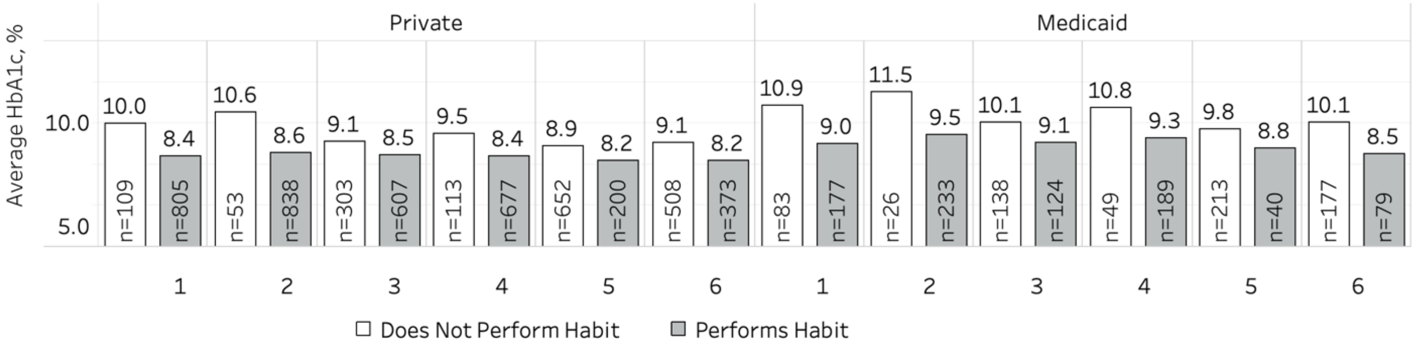

Supplemental Figure 1e - Average HbA1c for Individuals who Perform vs. Do Not Perform Habits 1 - 6 by Parent Education

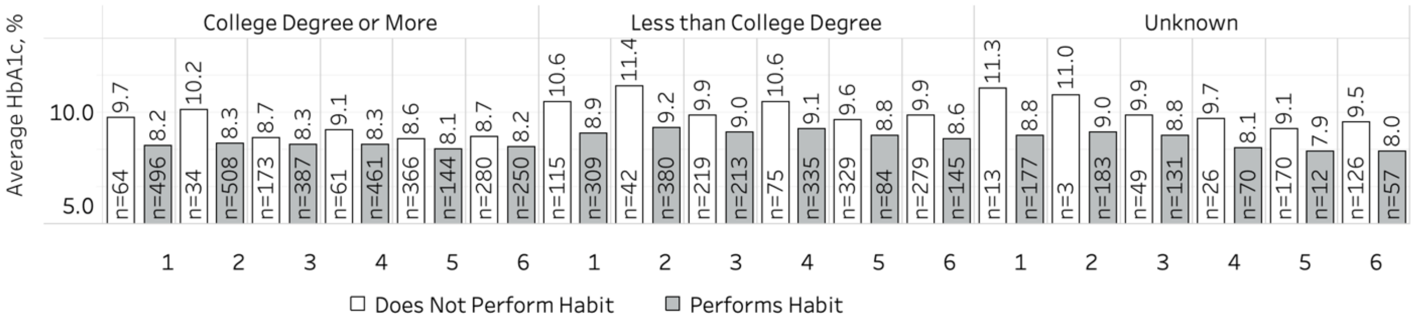

[a] The x axis represents habits 1-6. Instances of unknown habit performance were excluded from analysis.

© 2021 Lee JM et al. *JAMA Network Open.*

eFigure 2. Average HbA_{1c} Level and Time In Range (TIR) by Total Habit Score by Demographic Subgroups

Supplemental Figure 2a - Average HbA1c by Total Habit Score By Age Group

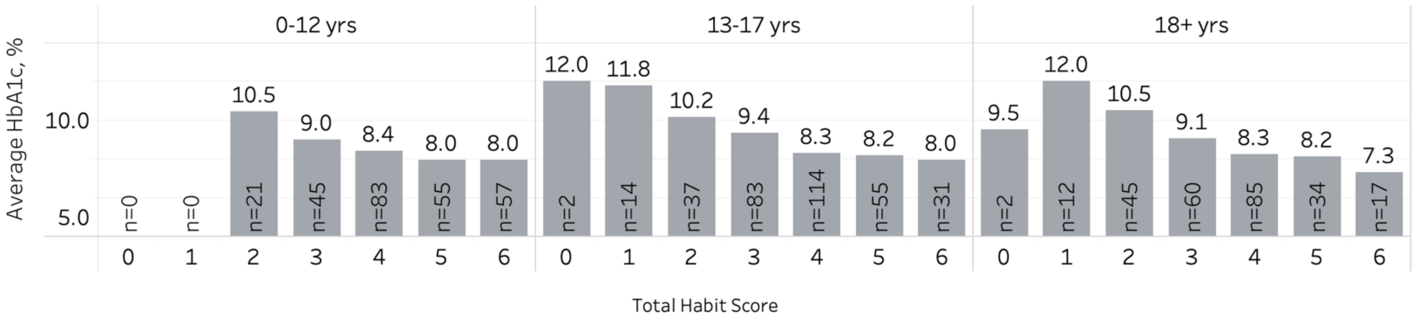

Supplemental Figure 2b - Average HbA1c by Total Habit Score By Sex

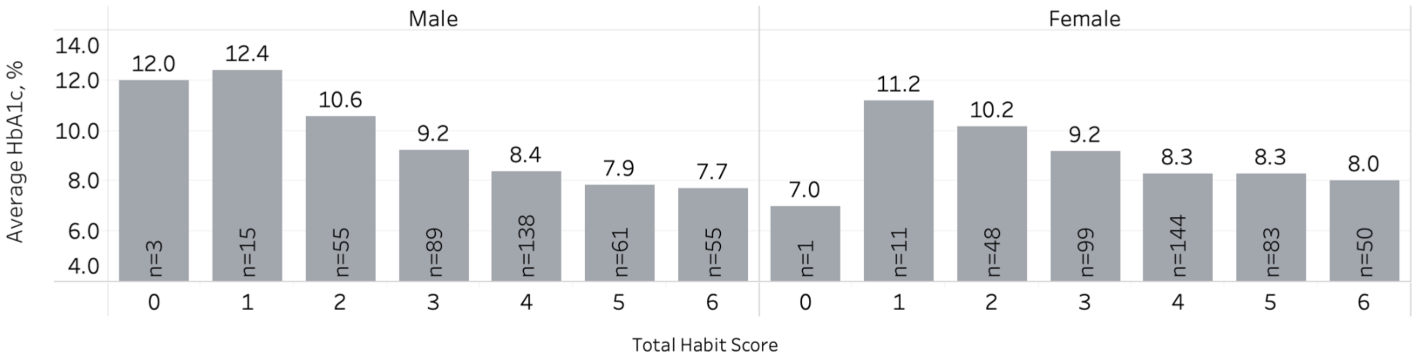

Supplemental Figure 2c - Average HbA1c by Total Habit Score By Race

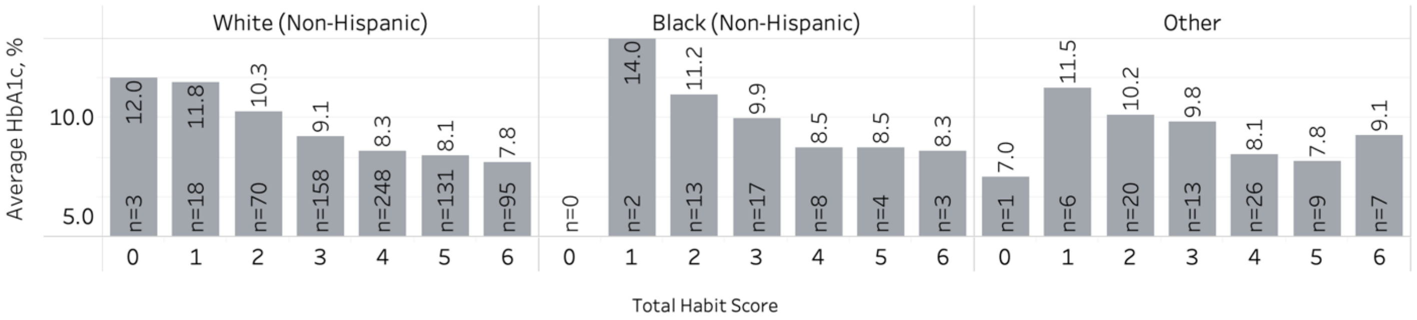

Supplemental Figure 2d - Average HbA1c by Total Habit Score By Insurance

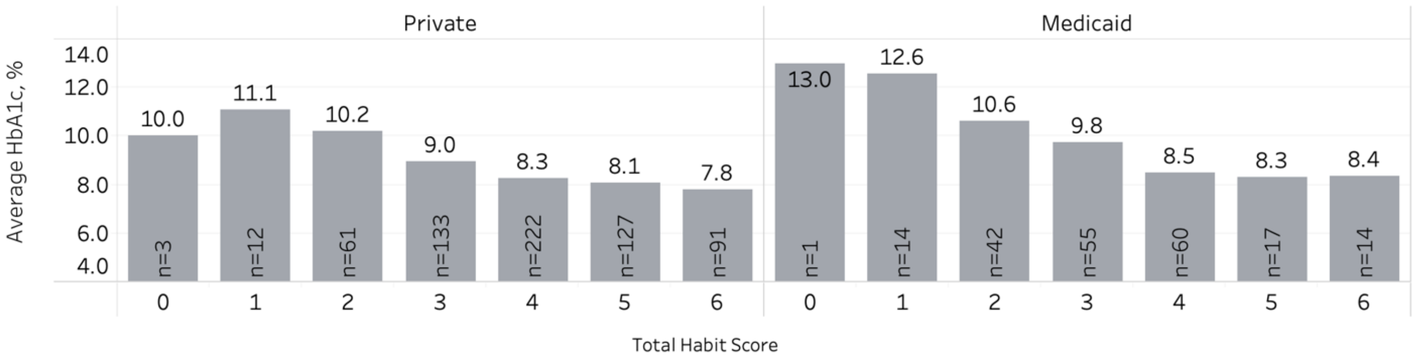

© 2021 Lee JM et al. *JAMA Network Open.*

Supplemental Figure 2e - Average HbA1c by Total Habit Score By Parent Education

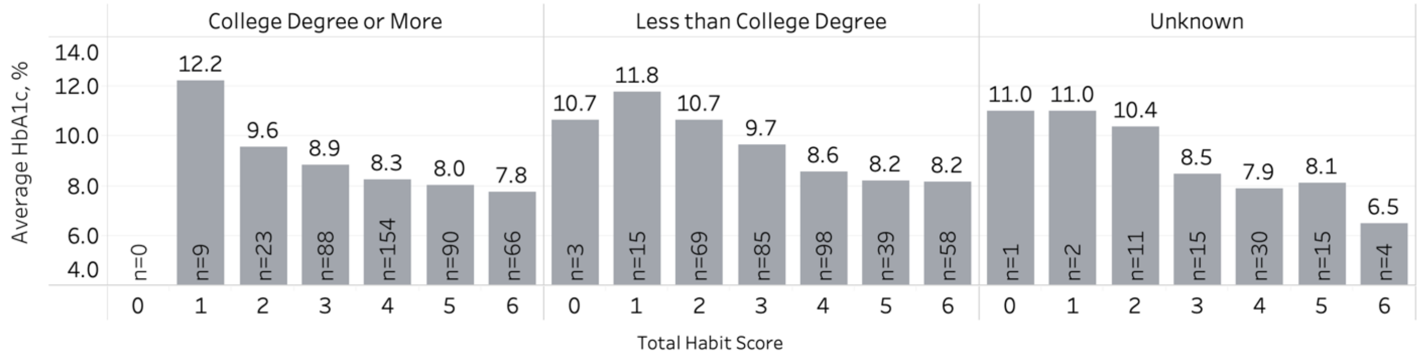

Supplemental Figure 2f - Average TIR by Total Habit Score By Age Group

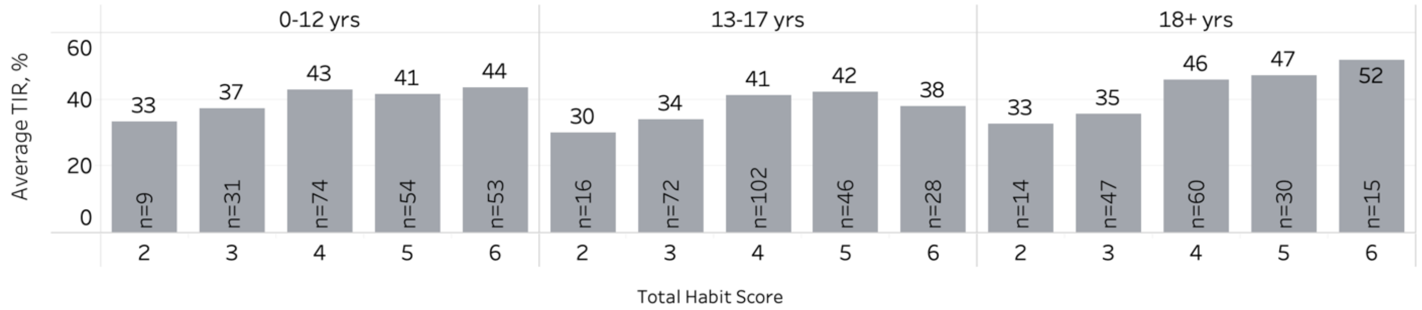

Supplemental Figure 2g - Average TIR by Total Habit Score By Sex

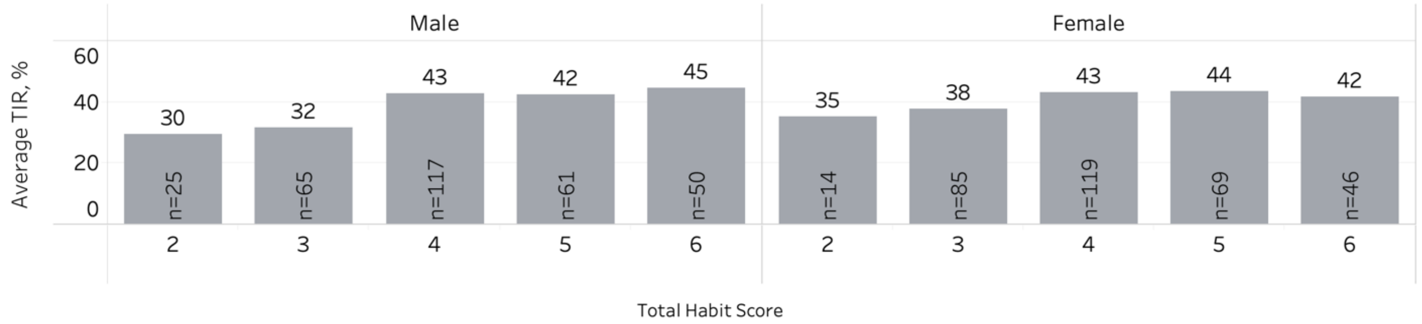

© 2021 Lee JM et al. *JAMA Network Open.*

Supplemental Figure 2h - Average TIR by Total Habit Score By Race

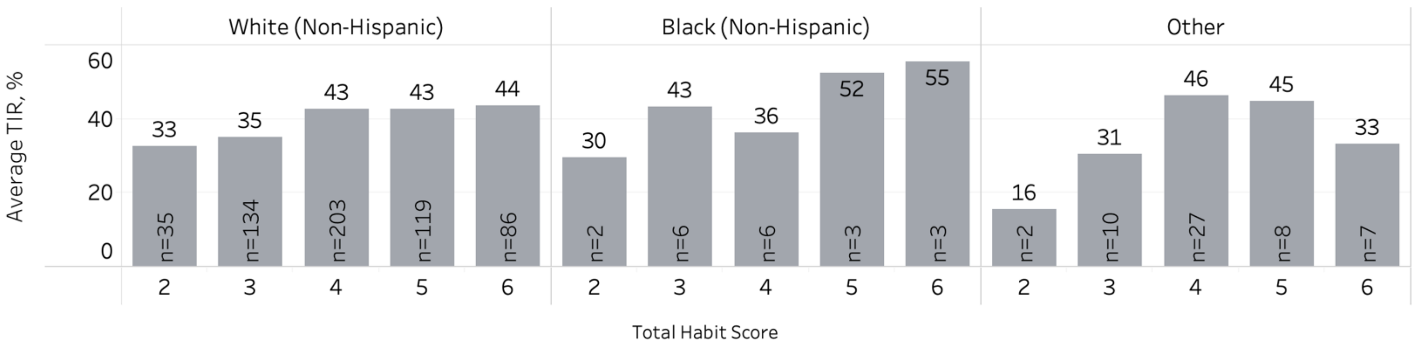

Supplemental Figure 2i - Average TIR by Total Habit Score By Insurance

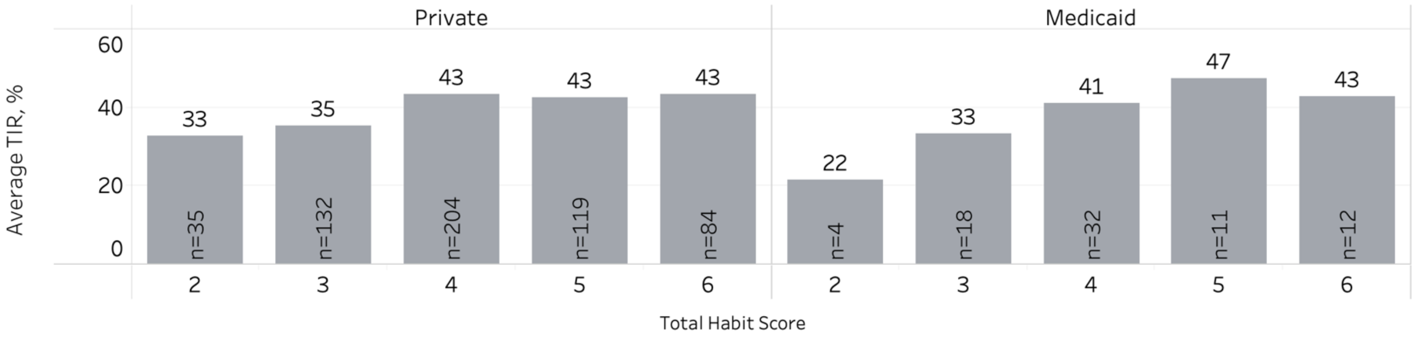

Supplemental Figure 2j - Average TIR by Total Habit Score By Parent Education

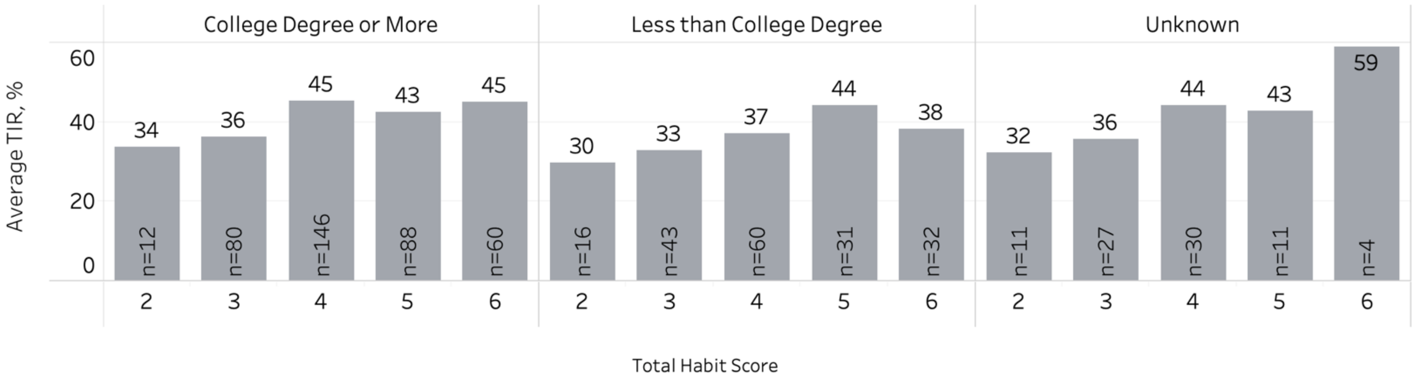

© 2021 Lee JM et al. *JAMA Network Open.*

eTable 3. Association of Demographic Variables Only, Habits Only, and Habits Plus Demographics with HbA_{1c} for 852 Patients with No Missing Habits and TIR for 506 Patients Using CGM with No Missing Habits

HbA1c for Patients with No Missing Habits									
	Demographics Only[a]			Habits Only[a]			Habits + Demographics[a]		
	Estimate	95% CI	*P* value	Estimate	95% CI	*P* value	Estimate	95% CI	*P* value
0-12 years	Ref						Ref		
13-17 years	0.41	(0.10, 0.71)	0.009				-0.15	(-0.43, 0.12)	0.28
18+ years	0.46	(0.14, 0.79)	0.005				-0.48	(-0.82, -0.15)	0.005
Male	Ref						Ref		
Female	-0.12	(-0.37, 0.14)	0.36				-0.06	(-0.27, 0.16)	0.61
White Non-Hispanic	Ref						Ref		
Black Non-Hispanic	1.31	(0.76, 1.85)	< 0.001				0.76	(0.28, 1.24)	0.002
Other	0.46	(0.04, 0.89)	0.03				0.11	(-0.26, 0.48)	0.57
Private Primary Insurance	Ref						Ref		
Medicaid	1.01	(0.72, 0.30)	< 0.001				0.54	(0.28, 0.80)	< 0.001
Parent with College Degree or More	Ref						Ref		
Parent with Less than College Degree	0.92	(0.66, 1.18)	< 0.001				0.45	(0.22, 0.68)	< 0.001
Unknown	0.02	(-0.42, 0.46)	0.92				-0.20	(-0.60, 0.19)	0.31
Does not Perform Habit 1				Ref			Ref		
Performs Habit 1				-1.80	(-2.10, -1.50)	< 0.001	-0.15	(-1.82, -1.20)	< 0.001
Does not Perform Habit 2				Ref			Ref		
Performs Habit 2				-2.34	(-2.82, -1.85)	< 0.001	-2.09	(-2.55, -1.62)	< 0.001
Does not Perform Habit 3				Ref			Ref		
Performs Habit 3				-1.02	(-1.28, -0.77)	< 0.001	-0.77	(-1.02, -0.51)	< 0.001
Does not Perform Habit 4				Ref			Ref		
Performs Habit 4				-1.13	(-1.46, -0.79)	< 0.001	-1.04	(-1.36, -0.71)	< 0.001
Does not Perform Habit 5				Ref			Ref		
Performs Habit 5				-0.61	(-0.80, -0.31)	< 0.001	-0.46	(-0.74, -0.17)	0.002
Does not Perform Habit 6				Ref			Ref		
Performs Habit 6				-0.85	(-1.11, -0.60)	< 0.001	-0.65	(-0.90, -0.40)	< 0.001
**Time in Range for CGM Users with No Missing Habits**									
	Demographics Only[a]			Habits Only[a]			Habits + Demographics[a]		
	Estimate	95% CI	*P* value	Estimate	95% CI	*P* value	Estimate	95% CI	*P* value
0-12 years	Ref						Ref		
13-17 years	-3.09	(-7.23, 1.05)	0.14				-1.20	(-5.52, 3.12)	0.58

© 2021 Lee JM et al. *JAMA Network Open.*

18+ years	2.50	(-2.38, 7.38)	0.32				5.09	(-0.43, 11.06)	0.07
Male	Ref						Ref		
Female	1.54	(-2.12, 5.19)	0.41				1.87	(-1.72, 5.47)	0.31
White Non-Hispanic	Ref						Ref		
Black Non-Hispanic	5.51	(-5.31, 16.33)	0.32				4.78	(-5.88, 15.44)	0.38
Other	-3.48	(-10.30, 3.35)	0.32				-2.73	(-9.35, 3.90)	0.42
Private Primary Insurance	Ref						Ref		
Medicaid	-0.81	(-6.28, 4.66)	0.77				-0.25	(-5.63, 5.13)	0.93
Parent with College Degree or More	Ref						Ref		
Parent with Less than College Degree	-5.48	(-9.49, -1.47)	0.007				-4.43	(-8.39, -0.48)	0.03
Unknown	1.39	(-5.87, 8.65)	0.71				0.80	(-6.47, 8.07)	0.82
Does not Perform Habit 2				Ref			Ref		
Performs Habit 2				16.73	(5.97, 27.50)	0.002	15.56	(4.83, 26.28)	0.005
Does not Perform Habit 3				Ref			Ref		
Performs Habit 3				5.62	(1.42, 9.82)	0.009	5.32	(1.07, 9.56)	0.01
Does not Perform Habit 4				Ref			Ref		
Performs Habit 4				11.02	(4.45, 16.00)	< 0.001	9.80	(4.04, 15.57)	< 0.001
Does not Perform Habit 5				Ref			Ref		
Performs Habit 5				1.59	(-2.37, 5.54)	0.43	1.58	(-2.41, 5.57)	0.44
Does not Perform Habit 6				Ref			Ref		
Performs Habit 6				2.42	(-1.27, 6.12)	0.20	2.50	(-5.05, 9.61)	0.19

[a] All models are adjusted for duration of diabetes

© 2021 Lee JM et al. *JAMA Network Open.*

eTable 4. Association of Demographic Variables Only, Habits Only, and Habits Plus Demographics with HbA_{1c} for 494 Patients Not Using CGM

HbA1c for Non-CGM Users									
	**Demographics Only**[a]			**Habits Only**[a]			**Habits + Demographics**[a]		
	**Estimate**	**95% CI**	***P* value**	**Estimate**	**95% CI**	***P* value**	**Estimate**	**95% CI**	***P* value**
0-12 years	Ref						Ref		
13-17 years	0.35	(-0.17, 0.88)	0.19				-0.08	(-0.63, 0.46)	0.76
18+ years	0.24	(-0.28, 0.75)	0.37				-0.41	(-1.01, 0.20)	0.19
Male	Ref						Ref		
Female	0.31	(-0.07, 0.68)	0.11				-0.28	(-0.67, 0.12)	0.17
White Non-Hispanic	Ref						Ref		
Black Non-Hispanic	0.12	(0.48, 1.77)	< 0.001				0.75	(0.07, 1.43)	0.03
Other	0.42	(-0.19, 0.98)	0.18				-0.13	(-0.76, 0.49)	0.68
Private Primary Insurance	Ref						Ref		
Medicaid	0.68	(0.29, 1.07)	< 0.001				-0.52	(0.11, 0.94)	0.01
Parent with College Degree or More	Ref						Ref		
Parent with Less than College Degree	0.77	(0.35, 1.20)	< 0.001				0.44	(-0.001, 0.88)	0.05
Unknown	0.43	(-0.12, 0.97)	0.12				-0.30	(-0.99, 0.40)	0.40
Does not Perform Habit 2				Ref			Ref		
Performs Habit 2				-1.55	(-2.12, -0.98)	< 0.001	-1.50	(-2.07, -0.92)	< 0.001
Does not Perform Habit 3				Ref			Ref		
Performs Habit 3				-0.72	(-1.11, 0.33)	< 0.001	-0.52	(-0.92, -0.12)	0.01
Does not Perform Habit 4				Ref			Ref		
Performs Habit 4				-1.10	(-1.57, -0.61)	< 0.001	-1.10	(-1.58, -0.62)	< 0.001
Does not Perform Habit 5				Ref			Ref		
Performs Habit 5				-0.47	(-1.07, 0.13)	0.13	-0.35	(-0.94, 0.25)	0.26
Does not Perform Habit 6				Ref			Ref		
Performs Habit 6				-1.06	(-1.48, -0.64)	< 0.001	-0.88	(-1.31, -0.44)	< 0.001

[a] All models are adjusted for duration of diabetes

© 2021 Lee JM et al. *JAMA Network Open.*

eTable 5. Association of Demographic Variables Only, Habits Only, and Habits Plus Demographics with HbA_{1c} for 749 Patients Using Pumps, Excluding Auto Mode Pumps

HbA1c for Pump Users (Excluding Auto Mode Pumps)									
	Demographics Only[a]			Habits Only[a]			Habits + Demographics[a]		
	Estimate	95% CI	*P* value	Estimate	95% CI	*P* value	Estimate	95% CI	*P* value
0-12 years	Ref						Ref		
13-17 years	0.58	(0.27, 0.89)	< 0.001				-0.16	(-0.44, 0.12)	0.26
18+ years	0.54	(0.22, 0.87)	< 0.001				-0.48	(-0.82, -0.14)	0.005
Male	Ref						Ref		
Female	-0.14	(-0.39, 0.11)	0.27				-0.11	(-0.33, 0.11)	0.33
White Non-Hispanic	Ref						Ref		
Black Non-Hispanic	1.29	(0.64, 1.93)	< 0.001				0.73	(-0.26, 1.21)	0.003
Other	-0.18	(-0.64, 0.27)	0.43				0.05	(-0.32, 0.42)	0.80
Private Primary Insurance	Ref						Ref		
Medicaid	0.78	(0.44, 1.11)	< 0.001				0.59	(0.33, 0.85)	< 0.001
Parent with College Degree or More	Ref						Ref		
Parent with Less than College Degree	0.75	(0.47, 1.03)	< 0.001				0.45	(0.22, 0.69)	< 0.001
Unknown	0.43	(0.09, 0.78)	0.01				-0.22	(-0.62, 0.17)	0.27
Does not Perform Habit 1				Ref			Ref		
Performs Habit 1				-1.63	(-1.90, -1.35)	< 0.001	-1.45	(-1.73, -1.16)	< 0.001
Does not Perform Habit 2				Ref			Ref		
Performs Habit 2				-2.02	(-2.44, -1.61)	< 0.001	-1.87	(-2.28, -1.46)	< 0.001
Does not Perform Habit 4				Ref			Ref		
Performs Habit 4				-1.03	(-1.34, -0.72)	< 0.001	-1.01	(-1.31, -0.71)	< 0.001
Does not Perform Habit 5				Ref			Ref		
Performs Habit 5				-0.51	(-0.79, -0.23)	< 0.001	-0.4	(-0.68, -0.12)	0.005
Does not Perform Habit 6				Ref			Ref		
Performs Habit 6				-0.97	(-1.20, -0.75)	< 0.001	-0.83	(-1.6, -0.61)	< 0.001

[a] All models are adjusted for duration of diabetes

© 2021 Lee JM et al. *JAMA Network Open.*

eTable 6. Association of Demographic Variables Only, Habits Only, and Habits Plus Demographics with HbA_{1c} for 692 Patients Using CGMs and Time in Range for 634 Patients Using CGMs, Adjusting for Flash vs Real-Time CGM

HbA1c for CGM Users[a] (Flash vs. Real-Time CGM Adjusted)									
	Demographics Only[b]			Habits Only[b]			Habits + Demographics[b]		
	Estimate	95% CI	*P* value	Estimate	95% CI	*P* value	Estimate	95% CI	*P* value
0-12 years	Ref						Ref		
13-17 years	0.23	(-0.04, 0.50)	0.09				-0.03	(-0.33, 0.26)	0.83
18+ years	-0.03	(-0.36, 0.30)	0.87				-0.37	(-0.74, 0.01)	0.06
Male	Ref						Ref		
Female	0.07	(-0.14, 0.29)	0.50				0.14	(-0.11, 0.38	0.27
White Non-Hispanic	Ref						Ref		
Black Non-Hispanic	0.42	(-0.24, 1.09)	0.21				0.22	(-0.54, 0.98)	0.57
Other	-0.08	(-0.47, 0.31)	0.69				0.29	(-0.15, 0.74)	0.20
Private Primary Insurance	Ref						Ref		
Medicaid	0.24	(-0.09, 0.57)	0.15				-0.38	(0.03, 0.74)	0.03
Parent with College Degree or More	Ref						Ref		
Parent with Less than College Degree	0.52	(0.27, 0.76)	< 0.001				0.42	(0.15, 0.69)	0.003
Unknown	0.07	(-0.26, 0.40)	0.68				-0.33	(-0.79, 0.13)	0.16
Does not Perform Habit 2				Ref			Ref		
Performs Habit 2				-1.46	(-2.09, -0.84)	< 0.001	-1.40	(-2.03, -0.77)	< 0.001
Does not Perform Habit 3				Ref			Ref		
Performs Habit 3				-0.41	(-0.67, -0.15)	0.002	-0.39	(-0.66, -0.13)	0.003
Does not Perform Habit 4				Ref			Ref		
Performs Habit 4				-0.70	(-1.05, -0.33)	< 0.001	-0.64	(-0.99, -0.28)	< 0.001
Does not Perform Habit 5				Ref			Ref		
Performs Habit 5				-0.25	(-0.52, 0.01)	0.06	-0.25	(-0.52, 0.01)	0.06
Does not Perform Habit 6				Ref			Ref		
Performs Habit 6				-0.5	(-0.72, -0.27)	< 0.001	-0.48	(-0.71, -0.25)	< 0.001
**Time in Range for CGM Users[a] (Flash vs. Real-Time CGM Adjusted)**									
	Demographics Only[b]			Habits Only[b]			Habits + Demographics[b]		
	Estimate	95% CI	*P* value	Estimate	95% CI	*P* value	Estimate	95% CI	*P* value
0-12 years	Ref						Ref		
13-17 years	-2.10	(-5.86, 1.67)	0.28				-0.88	(-5.27, 3.50)	0.69
18+ years	3.76	(-0.93, 8.45)	0.12				6.67	(1.04, 12.30)	0.02
Male	Ref						Ref		

© 2021 Lee JM et al. *JAMA Network Open.*

Female	1.19	(-1.93, 4.30)	0.46				1.41	(-2.27, 5.08)	0.45
White Non-Hispanic	Ref						Ref		
Black Non-Hispanic	2.83	(-6.31, 1.20)	0.54				4.49	(-6.58, 15.56)	0.43
Other	0.69	(-4.90, 6.27)	0.81				-2.86	(-9.58, 3.87)	0.40
Private Primary Insurance	Ref						Ref		
Medicaid	-1.25	(-6.07, 3.57)	0.61				-0.57	(-6.01, 4.87)	0.84

© 2021 Lee JM et al. *JAMA Network Open.*

eTable 6. Association of Demographic Variables Only, Habits Only, and Habits Plus Demographics with HbA_{1c} for 692 Patients Using CGMs and Time in Range for 634 Patients Using CGMs, Adjusting for Flash vs Real-Time CGM (*continued*)

Parent with College Degree or More	Ref						Ref		
Parent with Less than College Degree	-5.71	(-9.26, -2.15)	0.002				-5.11	(-9.17, -1.05)	0.01
Unknown	-0.13	(-5.00, 4.75)	0.96				1.52	(-5.77, 8.81)	0.68
Does not Perform Habit 2				Ref			Ref		
Performs Habit 2				1.26	(2.63, 12.36)	0.01	1.15	(1.52, 21.41)	0.02
Does not Perform Habit 3				Ref			Ref		
Performs Habit 3				3.37	(-0.34, 7.09)	0.07	4.28	(-0.28, 7.18)	0.07
Does not Perform Habit 4				Ref			Ref		
Performs Habit 4				8.29	(2.85, 13.73)	0.003	7.41	(2.16, 13.01)	0.006
Does not Perform Habit 5				Ref			Ref		
Performs Habit 5				1.49	(-2.17, 5.16)	0.42	1.64	(-2.09, 5.38)	0.39
Does not Perform Habit 6				Ref			Ref		
Performs Habit 6				3.89	(0.64, 7.13)	0.02	4.14	(0.89, 7.40)	0.01

[a] All models are adjusted for duration of diabetes

[b] Instances of unknown CGM type were excluded

© 2021 Lee JM et al. *JAMA Network Open.*
